# Spatial and temporal attention in developmental dyslexia

**DOI:** 10.3389/fnhum.2014.00331

**Published:** 2014-05-22

**Authors:** Milena Ruffino, Simone Gori, Daniela Boccardi, Massimo Molteni, Andrea Facoetti

**Affiliations:** ^1^Child Psychopathology Unit, Scientific Institute, IRCCS Eugenio MedeaLecco, Italy; ^2^Developmental and Cognitive Neuroscience Lab, Department of General Psychology, University of PaduaPadua, Italy

**Keywords:** spatial attention, temporal attention, temporal sampling, phonological decoding, reading disorder

## Abstract

Although the dominant view posits that developmental dyslexia (DD) arises from a deficit in phonological processing, emerging evidence suggest that DD could result from a more basic cross-modal letter-to-speech sound integration deficit. Letters have to be precisely selected from irrelevant and cluttering letters by rapid orienting of visual attention before the correct letter-to-speech sound integration applies. In the present study the time-course of spatial attention was investigated measuring target detection reaction times (RTs) in a cuing paradigm, while temporal attention was investigated by assessing impaired identification of the first of two sequentially presented masked visual objects. Spatial and temporal attention were slower in dyslexic children with a deficit in pseudoword reading (*N* = 14) compared to chronological age (*N* = 43) and to dyslexics without a deficit in pseudoword reading (*N* = 18), suggesting a direct link between visual attention efficiency and phonological decoding skills. Individual differences in these visual attention mechanisms were specifically related to pseudoword reading accuracy in dyslexics. The role of spatial and temporal attention in the graphemic parsing process might be related to a basic oscillatory “temporal sampling” dysfunction.

## Introduction

Developmental dyslexia (DD) is a neurodevelopmental disorder identified in about 10% of children which refers to a pattern of learning difficulties characterized by problems with accurate or fluent word recognition, poor decoding and poor spelling abilities, despite normal intelligence, and adequate access to conventional instruction (American Psychiatric Association, 2013).

According to the dual-route model (see Perry et al., 2007 for a review), written words can be processed either by the sub-lexical route, based on grapheme-to-phoneme correspondences, allowing us to read unfamiliar words and pseudowords, or by the lexical route, based on lexical unit correspondences, crucial for reading familiar and irregular words only. Phonological dyslexics show great difficulties in reading unfamiliar words and pseudowords compared to known words, and this is thought to arise from damage to the sub-lexical route. In contrast, surface dyslexia is characterized by impaired reading of irregular words, and this is thought to arise from a damage in the lexical route (e.g., Castles and Coltheart, 1993), potentially linked to an under-stimulation of the visual word recognition system resulting from low experience with literacy. However, in shallow orthographies such as Italian, spelling-sound irregularity is limited to the supra-segmental level (that is, to stress assignment). Thus, in Italian dyslexic children the increased weighting of sub-lexical processing does not permit precise measurement of the efficiency of the lexical-route (see also Gori et al., under revision). It is crucial to note that—regardless of spelling-sound regularity—for a beginning reader all words are at first pseudowords because the lexical-orthographic representations have still to be developed. Accordingly, most longitudinal studies have shown that beginning readers use primarily the sub-lexical route (see Sprenger-Charolles et al., 2003, for a review). Phonological decoding, which is typically measured by examining children's pseudoword reading performance, is one of the most critical skills for successful reading acquisition (e.g., Share, 1995). Interestingly, Ziegler et al. (2003) showed that dyslexics with both regular (German-speaking children) and irregular (English-speaking children) spelling-to-sound correspondences present an extremely slow and serial phonological decoding mechanism. Consequently, an efficient learning to read is crucially mediated by an accurate and fluent use of the sub-lexical route (e.g., Goswami et al., 2000; see Vellutino et al., 2004, for a review).

The underlying neurocognitive mechanisms that lead to the observed reading impairments are still hotly debated (see Vidyasagar and Pammer, 2010; Goswami, 2011). Impaired auditory and speech-sound processing is assumed to characterize the core deficit in DD (e.g., Tallal, 1980; Bradley and Bryant, 1983; Chandrasekaran et al., 2009; Vandermosten et al., 2010; Hornickel et al., 2012; see Wright et al., 2000; Goswami, 2003, 2011; Tallal, 2004; Gabrieli, 2009; Peterson and Pennington, 2012, for reviews). However, the hypothesis that DD arises specifically from a deficit of phonological awareness is still debated because of the circular relationship between reading ability and phonological skills acquisition (e.g., Blau et al., 2009; Dehaene et al., 2010; see Castles and Coltheart, 2004, for a review).

Emerging evidence suggested that DD could arise from a more basic cross-modal letter-to-speech sound integration deficit (e.g., Blau et al., 2009, 2010; Dehaene et al., 2010; see Blomert, 2011, for a recent review). A recent study has also shown that cross-modal binding is impaired at the very early stages of associative learning (Jones et al., 2013). Those authors suggested that dyslexic readers' difficulties in binding may be characterized by inadequate attentional deployment to spatial location. Letters have to be precisely selected from irrelevant and cluttering letters (Bouma, 1970; Bouma and Legein, 1977) by rapid orienting of visual attention (Yeshurun and Rashal, 2010) before the correct letter-to-speech sound integration applies (e.g., Hari and Renvall, 2001; Facoetti et al., 2010a; Vidyasagar and Pammer, 2010; Zorzi et al., 2012). Accordingly, recent studies have shown that visual attention is impaired not only in dyslexic children (e.g., Facoetti et al., 2010a; Lallier et al., 2010), but also in pre-readers at familial risk for DD. These results indicate that visual attention disorders are present before reading acquisition (e.g., Plaza and Cohen, 2007; Facoetti et al., 2010b) and that they are predictors of future reading acquisition skills controlling not only for age, IQ, and phonological processing, but also for non-alphabetic, visual-to-phonological mapping (Franceschini et al., 2012). Moreover, recent findings have shown that attentional training—not involving phonological or orthographic learning—by using action video games can improve reading abilities in children with DD (Franceschini et al., 2013). Visual attention can be oriented in space and time as a spotlight (i.e., attentional shifting; Posner, 1980; Yantis and Jonides, 1984; Jonides and Yantis, 1998). The spotlight of attention (i.e., attentional focus) can also be expanded or contracted in spatial extent to encompass large or small objects, respectively (e.g., Castiello and Umiltà, 1990; LaBerge, 1995; Ronconi et al., 2014). When attention is spatially concentrated in a small portion of the visual field it is called focused attention, while when it is spread across a large part of the visual field it is called distributed attention.

A specific relationship between non-linguistic deficits referred to as attentional shifting has been proposed by Hari and Renvall (2001). According to their multisensory “Sluggish Attentional Shifting” (SAS) framework, when dyslexics deal with rapid stimulus sequences, their automatic attention system cannot disengage fast enough from one item to the next one, yielding slow and degraded processing. SAS is assumed to distort cortical networks, more specifically those which support sub-lexical auditory-phonological (e.g., syllables and/or phonemes) and visual-orthographic (e.g., syllables and/or grapheme) representations. Attentional shifting and rapid processing deficits have been proposed as a more basic problem yielding to the phonological impairment observed in DD (e.g., Breznitz et al., 2013; see Farmer and Klein, 1995; Tallal, 2004, for reviews). This hypothesis is supported by a number of studies showing evidence for temporal processing of brief stimuli within both visual and auditory modalities in dyslexic populations (e.g., Hari and Kiesilä, 1996; Helenius et al., 1999; Hari et al., 1999, 2001; Renvall and Hari, 2002). Consequently, it has been suggested that non-linguistic deficits in dyslexics can be linked to a generally inefficient multi-sensory processing of perceptual stimuli (e.g., perceptual noise exclusion deficit; Sperling et al., 2005; Ziegler et al., 2009; Facoetti et al., 2010a) that impairs the ability to detect relevant stimuli (i.e., signals) when encountering signal interference induced by spatially (Geiger and Lettvin, 1987; Sperling et al., 2005; Geiger et al., 2008; Ruffino et al., 2010) or temporally close noise (Di Lollo et al., 1983; Visser et al., 2004; Montgomery et al., 2005; Facoetti et al., 2008). Notably, attentional deficits in children with DD, with specific language impairment and with autism spectrum disorder (e.g., Ronconi et al., 2012, 2013a) arise from a difficulty in the visual noise exclusion process that specifically requires more time between two stimuli to identify accurately the target as compared to typically developing children (e.g., Ruffino et al., 2010; Dispaldro et al., 2013; Ronconi et al., 2013b).

It is important to highlight that spatial attention is involved in perceptual noise exclusion (e.g., Carrasco et al., 2000, 2002, 2004), by optimizing the perceptual filter so that the signal is further processed and noise is excluded (Dosher and Lu, 2000). The major effect on perceptual functions is that spatial attention appears to enhance the neural representation of stimuli at the attended location (see Reynolds and Chelazzi, 2004, for a review). This signal enhancement manifests itself in a variety of ways, including faster reaction times (RTs) (Posner, 1980), improved sensitivity (lowered thresholds; Carrasco et al., 2002) and reduced interference exerted by flanking stimuli (Carrasco et al., 2000; Facoetti and Molteni, 2000; Boyer and Ro, 2007). An important unresolved issue is whether spatial attention can also speed up the rate at which information is processed. Spatial attention not only improves the spatial resolution, but also accelerates the rate of information processing (Carrasco and McElree, 2001). Moreover, it allows decisions to be based on information at the selected location alone, while disregarding any distracting stimuli (Dosher and Lu, 2000; Braun, 2002). On the basis of these perceptual effects, spatial attention influences all post-sensorial processes, such as the content of short-term memory, perceptual decisions and voluntary responses.

SAS may be a crucial factor behind difficulties in learning to read (Hari and Renvall, 2001; Facoetti et al., 2005) and may be one important factor involved in perceptual difficulties, mostly in tasks requiring an efficient noise exclusion mechanism. Moreover, spatial attention deficits have been repeatedly shown in DD (e.g., Cestnick and Coltheart, 1999; Facoetti et al., 2005, 2006; Bosse et al., 2007; see Hari and Renvall, 2001; Vidyasagar and Pammer, 2010, for reviews) and more specifically in dyslexics with poor pseudoword reading ability (Cestnick and Coltheart, 1999; Buchholz and McKone, 2004; Facoetti et al., 2006, 2008; Roach and Hogben, 2007; Jones et al., 2008; Ruffino et al., 2010). The efficient learning of sub-lexical spelling-sound mappings requires not only accurate representations at the phoneme or syllabic level (Snowling, 2000; Goswami, 2003, respectively), but also an efficient graphemic parsing mechanism (Facoetti et al., 2006, 2010a; Perry et al., 2007; Ruffino et al., 2010; Vidyasagar and Pammer, 2010). These visual attentional processes are hypothesized to be crucially involved in spelling-to-sound conversion mechanisms. Computational models of silent or oral reading assume that graphemic parsing requires the serial engagement of visual attention onto, and its disengagement from, each sub-lexical unit. Among the processes necessary for adequate processing along the sub-lexical route, a graphemic parsing mechanism may be critically linked to the selection mechanism of visual attention (Zorzi, 2005; Perry et al., 2007; Zorzi et al., 2012; Schneps et al., 2013a,b).

Although it has already been demonstrated that visual spatial and temporal attention deficits could contribute independently to the poor reading outcome of dyslexic individuals, as yet, no studies have shown that both spatial and temporal attentional deficits co-occur in the same group of children with DD. These findings indicate that a sluggish shifting of spatial attention is specifically related to a perceptual noise exclusion deficit in DD.

Thus, in the current study, we investigated whether both spatial and temporal attention are impaired in DD with poor phonological decoding, and if they have a specific predictive relationship with phonological decoding skill.

We measured the time-course of visual spatial attention (VSA) and visual temporal attention (VTA) in two groups of dyslexic children, classified on the basis of their phonological decoding (dis)ability, and one group of controls matched for chronological age and IQ.

**Figure 1:**
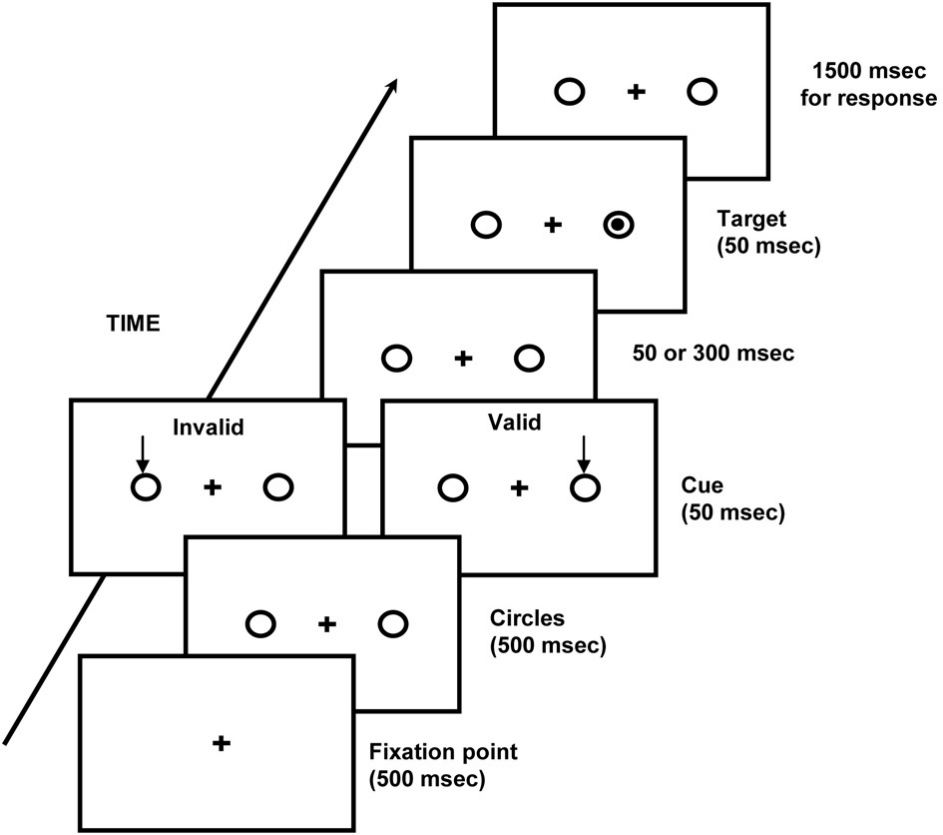
**Schematic representation of the display used in the visual spatial attention task**.

VSA has been extensively studied by using spatial cuing paradigms (Posner, 1980), in which covert attention (without eye movements) is engaged across two locations of a forthcoming target stimulus by a peripheral, informative spatial cue (i.e., cue location predicts target location) at two variable cue-target intervals (100 and 350 ms). Stimuli presented at the valid location are detected faster than stimuli appearing at the invalid location (the cuing effect reflects facilitation and inhibitory mechanisms of attention; see Figure 1). These attentional effects have been interpreted as a consequence of enhanced sensory processing of stimuli appearing at attended locations (Posner, 1980; Carrasco et al., 2000, 2002, 2004), and indicate that VSA has been efficiently engaged. Processing facilitation in VSA is usually found at short cue-target delays only (e.g., 50–150 ms; see Klein, 2000, for a review; see also Facoetti et al., 2010a). Therefore, sluggish VSA might be revealed by a delay in the normal time-course of VSA, i.e., this attentional processing facilitation should be present at longer but not at shorter cue-target delays.

**Figure 2:**
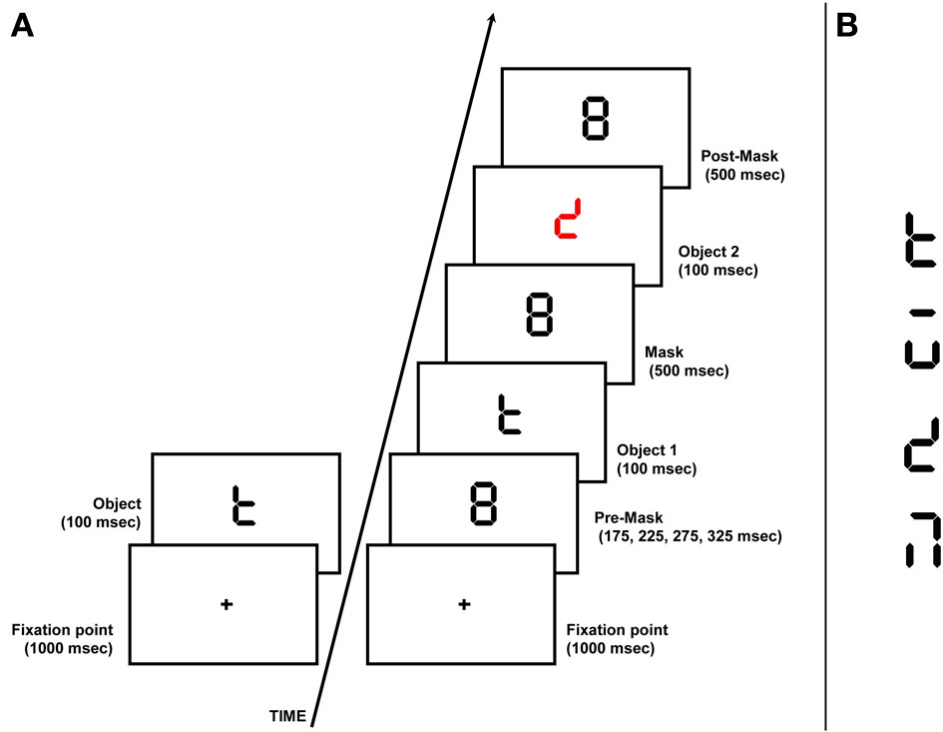
**Schematic representation of the display used in the visual temporal attention task **(A)**; Stimuli used in the visual temporal attention task **(B)****.

VTA was measured by using an identification task in which the first of two sequentially and centrally presented, forward and backward masked objects had to be recognized (i.e., signal + noise condition; Duncan et al., 1994), and it was compared to the identification of a single displayed object (signal condition). The first visual object (O1) preceded the onset of the second visual object (O2), by a short stimulus-onset-asynchrony (150 ms O1–O2 SOA; Facoetti et al., 2008). However, in order to highlight the O2 perceptual segregation and simplify the task for children, it was displayed in a different color from O1 (see Figure 2A). The accuracy to identify O1 allowed us to measure the efficiency of temporal engagement onto a centrally presented visual object.

## Materials and methods

### Participants

Participants were 32 Italian children with DD recruited at the Child Psychopathology Unit, Scientific Institute, IRCCS Eugenio Medea, Bosisio Parini, Lecco. Chronological age ranged from 8 to 14 (mean = 10, *SD* = 1.85). Reading performance, in terms of accuracy and/or speed, was 2 SDs below the norm on at least one of the age-standardized Italian tests included in the clinical battery (single word and pseudoword reading; Sartori et al., 1995). Dyslexics were selected on the basis of: (i) a full scale IQ greater than 85, as measured by the Wechsler Intelligence Scale for Children-Revised (WISC-R, Wechsler, 1993); (ii) normal or corrected-to-normal vision and hearing; (iii) the absence of neurological and/or psychiatric disorders; and (iv) the absence of attention deficit disorder with or without hyperactivity (because of its high co-morbidity with DD), as evaluated through DSM-IV diagnostic criteria (American Psychiatric Association, 1994). None of the dyslexic children followed an intensive nor a specific training based on well-documented efficacy. Forty-three typically reading children (NR), matched on age and IQ, were also selected. They were aged between 7 and 14 years (mean = 10, *SD* = 2.31) and were recommended as typical readers by their parents, confirmed by individual evaluation in a quiet room in their school. Reading performance was considered typical when accuracy and speed were higher than 1 SD below the norm of the age-standardized Italian tests included in the clinical battery (single word and pseudoword reading task; Sartori et al., 1995). Informed written consent was obtained from the parents of each child, and the Scientific Institute, IRCCS “Eugenio Medea” ethics committee approved the research protocol. The entire research process was conducted according to the principles expressed in the Declaration of Helsinki. Dyslexic and normally reading children were comparable for chronological age (two-tailed *t*-test, *p* = 0.96) and Performance IQ (Figure Completion subtest, WISC-R, Wechsler, 1993; two-tailed *t*-test, *p* = 0.09), but they were significantly different for both accuracy and speed of word and pseudoword reading (two-tailed *t*-test, all *p*s < 0.0001).

### Dyslexia sub typing

In order to study the sub-lexical route efficiency, dyslexic children were divided into two groups on the basis of their accuracy in phonological decoding. In particular, their ability to read aloud was measured on a list of 50 Italian regular and 50 Italian irregular^1^ words and 50 pseudowords, in order to assess phonological decoding ability. We measured only accuracy in our study because low accuracy is frequently a problem observed even in Italian dyslexics (e.g., Facoetti et al., 2006, 2010a; Ruffino et al., 2010). Specifically, a dyslexic child was assigned to the DDP− group (where P- indicates severe pseudoword reading) if pseudoword reading accuracy was below the 5th percentile in comparison to normally reading children. We selected this criterion in order to find an inefficient sub-lexical route. All dyslexic children who did not meet the criterion for inclusion in the DDP− group were assigned to the DDP+ group (where P+ indicates less severe pseudoword reading). The percentage mean of pseudoword reading accuracy was 64% (*SD* = 10.14) for the DDP− and 79% (*SD* = 14.83) for the DDP+ group (*p* < 0.0001).

### Phonological tasks

We administered a Pseudowords Phoneme Blending (PPB) task and a Pseudoword Short-Term Memory (PSTM) task to the participants.

In the PPB task, single phonemes were presented, and participants were asked to pronounce the resulting pseudowords from their synthesis (i.e., G-A-S-T-I-B-O = GASTIBO). Performances were calculated on the basis of the number of pseudowords correctly pronounced (the number of words administered were one for familiarization, nine experimental; the number of phonemes included in each pseudoword ranged from 7 to 10). The PSTM task consisted of repeating lists of pseudoword trigrams orally presented (i.e., two items ranging from 2 to 8 trigrams) in the same order as originally presented. Performances were indexed as the number of phonemes correctly repeated in the correct position (the maximum score was 210 phonemes). The number of list items increased with correct responses until participants made an error in both list items administered of the same length. For additional details see Supplementary Material.

### Visual attentional tasks

#### Spatial attention

Testing was carried out in a dimly lit (luminance of 1.5 cd/m^2^) and quiet room (approximately 50 dB SPL). Participants were seated in front of a computer screen (background luminance of 0.5 cd/m^2^), with their head positioned on a chinrest so that the eye-screen distance was 40 cm. Stimuli were white on a black background and had a luminance of 24 cd/m^2^. Each trial started with the onset of the fixation point (1° visual angle; 500 ms). Two circles (2.5°) were displayed peripherally (8° eccentricity, one to the left and one to the right of the fixation point) and 500 ms later the visual cue was shown, consisting of an arrow (1.5° visual angle) displayed for 50 ms above one of the circles. In response trials, a target (dot, 0.5°; duration 50 ms) was presented after one of two cue-target stimulus onset asynchronies (SOA, 100 or 350 ms) in one of the two possible locations. The probability that the cue was presented in the target location was about 80% (i.e., the cue location was predictive of target location). In contrast, in catch trials the target was not presented and participants did not have to respond. Catch trials were intermixed with response trials. Participants were instructed to react as quickly as possible to the onset of the visual targets by pressing the spacebar on the computer keyboard (detection task). Simple RTs and error rates were recorded by the computer. The maximum time allowed to respond was 1500 ms. The inter-trial interval was 1000 ms, after that time the trial started automatically. The experimental session consisted of 128 trials divided into two blocks of 64 trials each, which were distributed as follows: 40 valid trials (20 targets in the right visual field and 20 in the left visual field, 10 for each SOA), 12 invalid trials (6 targets in the right visual field and 6 in the left visual field, 3 for each SOA), and 12 catch trials (6 for each SOA; see Figure 1).

#### Temporal attention

The experimental environment was the same as described above for the spatial attention task. Each trial began with the onset of the fixation mark (0.3° of visual angle; duration 500 ms). Participants were instructed to keep their eyes on the fixation mark throughout the duration of the trial. Two conditions, a “signal alone” (O) and “signal + noise” (O+noise), were randomly presented to each participant. In the O condition a single object (duration 100 ms) was displayed and the aim was to measure the participants' ability to identify the experimental stimuli. In the O+noise condition an 8 digital clock-face font comprising seven line segments was displayed for a variable time exposure (175, 225, 275, or 325 ms) acting as a pre-mask, two successive objects (black O1 and red O2) were presented for 100 ms by removing some of the line segments (see Figure 2B, for stimuli used), each followed by a post-mask (8-digital clock-face font) of different duration (i.e., for O1 = 50 and for O2 = 500 ms). This condition was designed to maximize the load of the perceptual noise exclusion mechanism. All visual stimuli displayed had a luminance of 0.6 cd/m^2^, the background appeared white and had a luminance of 119 cd/m^2^. Participants viewed the sequence of stimuli binocularly and they were instructed to identify, at the end of each trial, O and O1 as accurately as possible, measuring the attentional engagement onto the target (O and O1).

Before the start of the experimental session, participants viewed each of the four different stimuli one by one with no time constraint (familiarization phase). After each trial all four possible targets were presented on the screen together (two targets per line). Participants responded by pointing on the screen. These responses were registered by the experimenter by pressing the corresponding key on a computer keyboard and no feedback was provided. The experimental session consisted of 40 trials (16 for the O condition and 24 for the O+noise condition; see Figure 2).

## Results

### Age, IQ, reading, and phonological skills: groups analysis

The differences between the three groups in age, Performance IQ, experimental reading paradigm (the accuracy in regular, irregular word, and pseudoword reading) and phonological tasks (pseudowords and phonemes correctly reported in the PPB and PSTM task, respectively) were analyzed. Results showed no significant differences in age or Performance IQ [*F* < 1 and *F*_(1, 72)_ = 1.94, *p* > 0.05, respectively], whereas significant differences were shown in all reading indexes [Regular words: *F*_(1, 72)_ = 9.77, *p* < 0.0001; Irregular words: *F*_(1, 72)_ = 9.86, *p* < 0.0001; and Pseudowords: *F*_(1, 72)_ = 54.14, *p* < 0.0001] and in the two phonological tasks. The NR group demonstrated a significantly higher number of correctly pronounced pseudowords in the PPB task [*F*_(1, 72)_ = 15.08, *p* < 0.0001] and correctly pronounced phonemes in the PSTM task [*F*_(1, 72)_ = 12.31, *p* < 0.0001] compared to the two groups of dyslexic children. Planned comparisons demonstrated that, although both dyslexic groups were significantly different from NR in all reading and phonological abilities, DDP− and DDP+ were different only in pseudoword reading accuracy (see Table 1). Thus, the selective deficit in phonological decoding skills observed in the DDP− group is difficult to explain with respect to differences in their phonological processing, which did not significantly differ between the two dyslexic groups (see Table 1).

**Table 1:** **Mean (M) and standard deviation (SD) of age (months), Performance IQ (Figure Completion, Wechsler, 1993), Reading abilities (Regular, Irregular words, and Pseudowords), pseudowords phoneme blending (number of correct pseudowords), and a pseudoword short-term memory (number of correct phonemes) in normally reading children (NR) and developmental dyslexics without (DDP+) and with (DDP−) phonological decoding deficit**.

	**NR (*N* = 43)**		**DDP+ (*N* = 18)**		**DDP− (*N* = 14)**		**Comparison NR vs. DDP +**			**Comparison NR vs. DDP −**			**Comparison DDP+ vs. DDP−**		
	***M***	***SD***	***M***	***SD***	***M***	***SD***	***t*_(59)_**	***P***	***C.'s d***	***t*_(55)_**	***P***	***C.'s d***	***t*_(30)_**	***P***	***C.'s d***
Age (months)	122.23	27.87	122.50	20.00	122.57	25.45	−0.04	>0.05	−0.01	−0.04	>0.05	−0.01	0.01	>0.05	−0.003
Performance IQ (ss)	13.65	2.61	12.11	3.41	13.07	2.43	1.17	>0.05	0.51	0.76	>0.05	0.23	−0.93	>0.05	−0.32
Regular words reading (%)	99.67	0.75	90.56	15.76	93.57	3.16	2.45	**<0.05**	0.82	7.17	**<0.001**	2.66	−0.80	>0.05	−0.26
Irregular words reading (%)	98.28	2.64	88.94	16.77	89.00	7.51	2.35	**<0.05**	0.78	4.53	**<0.001**	1.65	−0.01	>0.05	−0.005
Pseudowords reading (%)	93.77	6.02	78.78	14.83	64.43	10.14	4.15	**<0.002**	1.32	10.25	**<0.001**	3.52	−3.24	**<0.001**	1.13
Number of correct pseudowords	5.44	1.76	3.50	2.75	2.18	1.99	2.77	**<0.02**	0.84	5.46	**<0.001**	1.73	−1.57	>0.05	0.55
Number of correct phonemes	56.74	17.20	40.61	15.41	35.86	11.97	3.60	**<0.002**	0.99	5.05	**<0.001**	1.41	−0.98	>0.05	0.34

*The effect size (Cohen's d) is reported as C.'s d*.

### Visual attentional tasks: groups analysis

#### Spatial attention

**Figure 3:**
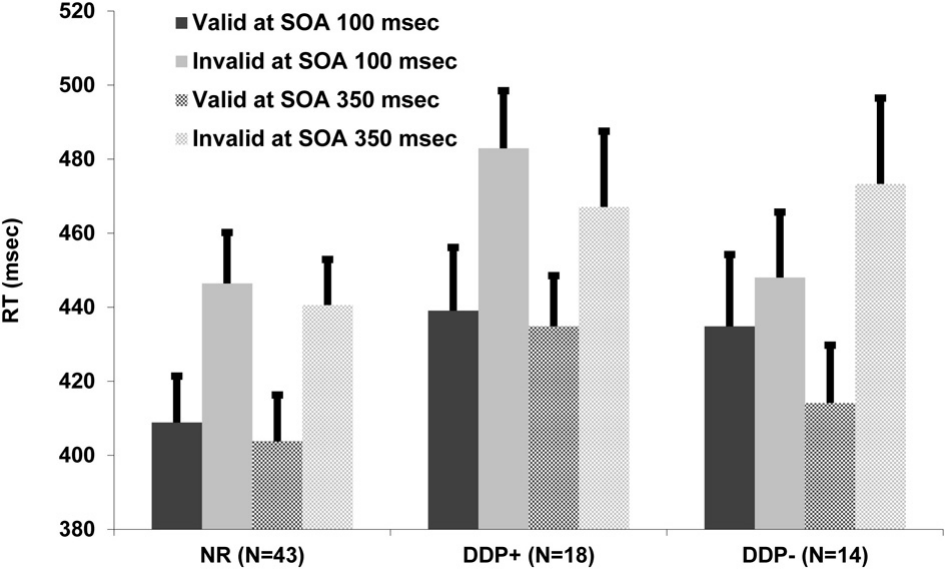
**Mean RT and standard error as a function of group (NR, DDP+, and DDP−), target condition (valid vs. invalid cue), and cue-target SOA (100 and 350 ms)**.

**Figure 4:**
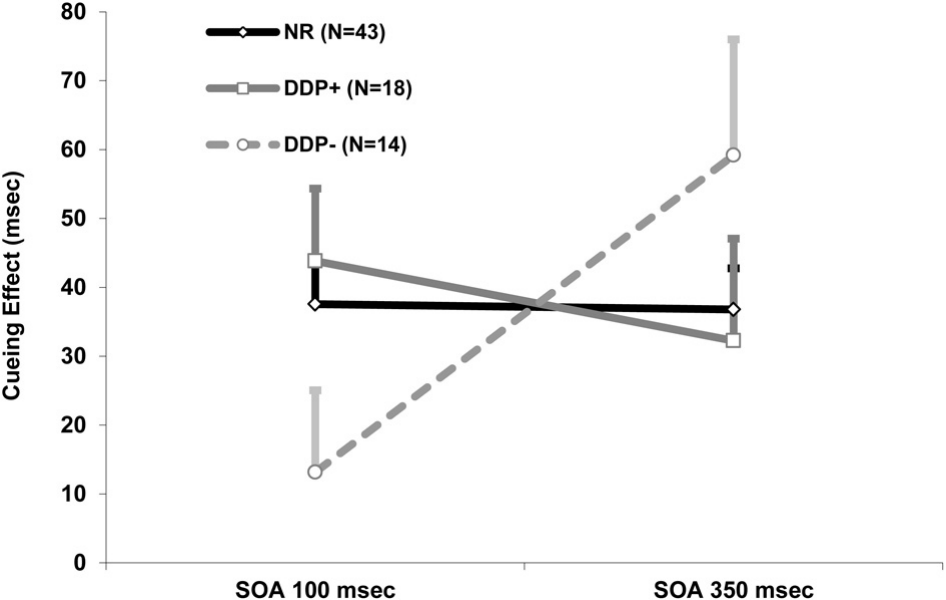
**Mean of cuing effect (i.e., invalid – valid RT differences) and standard error as a function of cue-target stimulus onset asynchrony (SOA; 100 and 350 ms) and group (NR, DDP+, and DDP−)**.

Mean correct detection RTs were analyzed with a mixed ANOVA that had target condition (valid and invalid) and SOA (100 and 350 ms) as within-subject factors, and group (NR, DDP+, and DDP−) as between-subject factor. The target condition main effect was significant, *F*_(1, 72)_ = 69.85, *p* < 0.0001; RTs were slower in the invalid condition (460 ms) than in the valid condition (423 ms; cuing effect = 37 ms). No other main effects were significant. Notably, the critical three-way interaction group × SOA × target condition interaction was significant, *F*_(2, 72)_ = 3.77, *p* < 0.05 (see Figure 3), indicating a different time-course of attentional orienting in the three groups. At short SOA, both dyslexic groups, in the valid condition, appear to detect targets more slowly in comparison to the normal readers. These results show an apparently reduced facilitation effect in both dyslexic groups. In the invalid condition the DDP− group was similar to the NR group suggesting an unimpaired inhibition mechanism in the DDP− group. The DDP− group was faster than the DDP+ in the invalid cue condition suggesting an abnormal inhibition mechanism in the DDP+ group. All three groups showed a significant cuing effect (i.e., invalid-valid RT differences) at 350 ms SOA [NR = 37 ms (*SD* = 39.26), DDP+ = 32 ms (*SD* = 67.74), and DDP− = 59 ms (*SD* = 62.91); all *p*s < 0.005], demonstrating that they are able to orient spatial attention at the longer time interval. In contrast, only the DDP− group did not show a significant cuing effect at the 100 ms SOA [NR = 37 ms (*SD* = 38.15), DDP+ = 32 ms (*SD* = 44.50), and DDP− = 13 ms (*SD* = 44.51)], demonstrating a sluggish VSA in DDP− in comparison to NR and DDP+ (see Table 2). Moreover, the DDP− group showed and amplified cuing effect in comparison to NR and DDP+ grouped together at 350 ms SOA (*p*<0.05; see Figure 4).

**Table 2:** **Mean (M) and standard deviation (SD) of cuing effect at 100 and 350 ms cue-target delay**.

	**NR (*N* = 43)**		**DDP+ (*N* = 18)**		**DDP− (*N* = 14)**		**Comparison NR vs. DDP +**			**Comparison NR vs. DDP −**			**Comparison DDP + vs. DDP−**		
	***M***	***SD***	***M***	***SD***	***M***	***SD***	***t*_(59)_**	***P***	***C.'s d***	***t*_(55)_**	***P***	***C.'s d***	***t*_(30)_**	***P***	***C.'s d***
100 ms cue-target delay	37.56	38.15	43.83	44.50	12.74	44.51	−0.52	>0.05	−0.01	2.03	**<0.05**	0.88	1.96	**<0.05**	0.96
350 ms cue-target delay	36.79	39.26	32.25	67.74	59.19	62.91	0.27	>0.05	0.08	−1.26	>0.05	−0.43	−1.15	>0.05	−0.41

*The Effect Size (Cohen's d) is reported as C.'s d*.

In summary, the data highlighted a marked offset of the time-course of visual attention in DDP−, which suggests a sluggish VSA, because differences were selectively present only for the shorter SOA. DDP− group show that for the longer SOA, VSA was abnormally oriented.

#### Temporal attention

**Figure 5:**
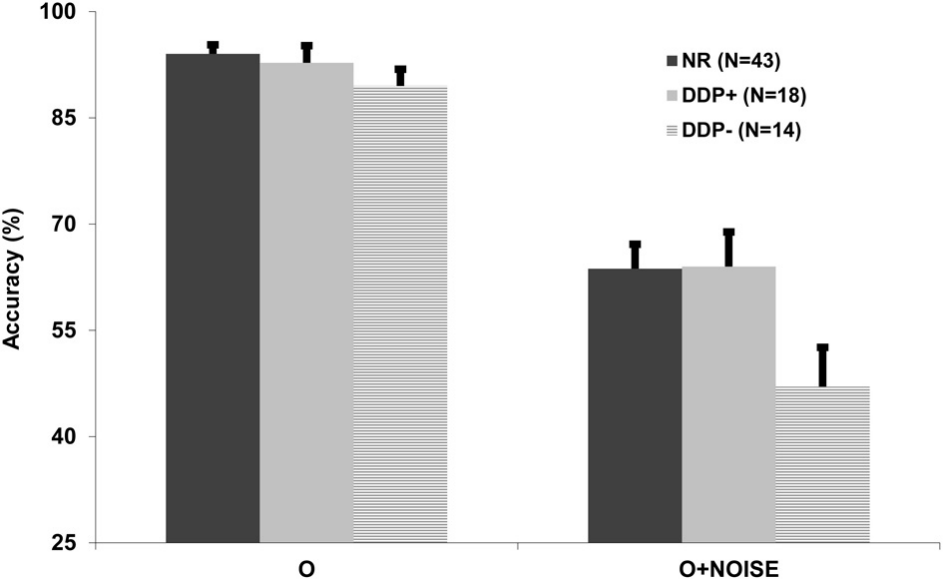
**Mean O and standard error as a function of group (NR, DDP+, and DDP−), signal accuracy (O), and signal + noise accuracy (O+noise)**.

The identification accuracy mean in the O condition was analyzed by a One-Way ANOVA with Group as the between subjects factor. The group main effect was not significant [*F*_(2, 72)_ = 1.34, *p* > 0.05], highlighting that signal identification in the DDP− group did not differ from either the NR or DDP+ groups. In contrast, the mean O1 accuracy rate^2^ ANOVA showed a significant group effect [*F*_(2, 72)_ = 3.23, *p* < 0.05], demonstrating that signal identification in presence of noise was significantly impaired in DDP− (47%), in comparison to NR (64%) and DDP+ (64%). Planned comparisons showed that the DDP− group, as compared to NR [*t*_(72)_ = 5.93, *p* < 0.005] and DDP+ [*t*_(72)_ = 4.84, *p* < 0.05], was significantly impaired in signal identification when presented with noise (see Table 3). In order to test perceptual-noise exclusion mechanism, “intrusion” errors for the identity of the second target were analyzed by a 1-tailed Independent Sample Test, showing a higher incidence of identity intrusions in the DDP− group (mean = 42%, *SD* = 11.22) compared to NR (mean = 36%, *SD* = 12.19), *p* < 0.05 and DDP+ (mean = 34%, *SD* = 10.97), *p* < 0.05 (details are reported in Table 4). In summary, our results showed that only dyslexics with phonological decoding deficit present difficulties in their perceptual-noise exclusion mechanism (see Figure 5).

**Table 3:** **Mean (M) and standard deviation (SD) of single object (O) and signal+noise (O+noise)**.

	**NR (*N* = 43)**		**DDP+ (*N* = 18)**		**DDP− (*N* = 14)**	
	***M***	***SD***	***M***	***SD***	***M***	***SD***
O	94	8.27	93	10.53	89.50	9.30
O+noise	64	21.89	64	21.66	47	23.83

**Table 4:** **Mean (M) and standard deviation (SD) of “intrusion” errors for the identity of the second target in the temporal attention task (O+noise condition)**.

	**NR (*N* = 43)**		**DDP+ (*N* = 18)**		**DDP− (*N* = 14)**		**Comparison NR vs. DDP+**					**Comparison NR vs. DDP−**					**Comparison DDP+ vs. DDP**				
	***M***	***SD***	***M***	***SD***	***M***	***SD***	***t*_(59)_**	***P***	***C.'s d***	***l.C.L***	***u.C.L***.	***t*_(55)_**	***P***	***C.'s d***	***l.C.L***	***u.C.L***.	***t*_(30)_**	***P***	***C.'s d***	***l.C.L***	***u.C.L***.
“intrusion” errors (O+noise condition)	35.81	12.19	34.44	10.97	42.14	11.22	0.43	>0.05	0.003	−5.3	8.03	−1.79	**<0.05**	0.051	*−13.71*	1.05	−1.94	**<0.05**	0.113	*−15.76*	0.36

*The effect size (Cohen's d) is reported as C.'s d. The confidence limits are reported as l.C.L (lower Confidence Limit), u.C.L (upper Confidence Limit)*.

### Relationship between visual attention and phonological decoding in dyslexic children

Our results demonstrate a specific VSA and VTA deficit in the DDP− group. In order to investigate a possible relationship between individual measures of the cuing effect (VSA) time-course, the perceptual noise exclusion mechanism (VTA), and phonological decoding skill across our entire sample of dyslexic children (*N* = 32), bivariate correlations were computed. Reading abilities were measured on regular words, irregular words and pseudowords. The time-course of VSA was indexed by the difference between the cuing effects at 350 and 100 ms SOA. The efficiency of the VTA corresponded to the identification accuracy mean in the O+noise condition. Phonological abilities were measured by using the efficiency in the PPB and PSTM tasks.

**Figure 6:**
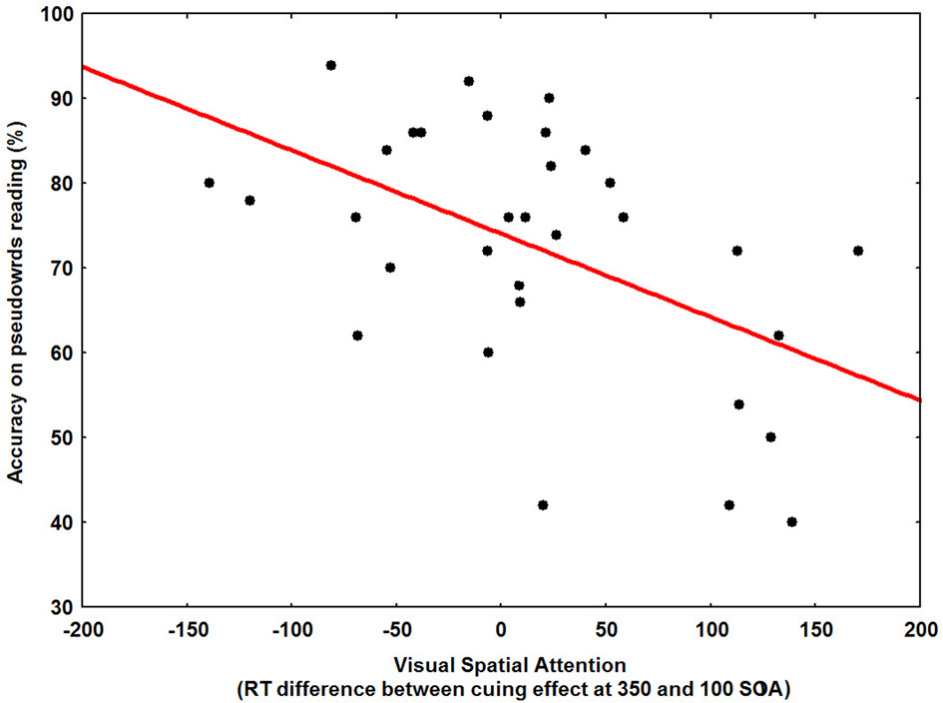
**Scatter plot of the relationship between time-course of visual spatial attention (RT difference between cuing effect at 350 and 100 ms SOA) and pseudoword reading accuracy across our entire sample of dyslexic children (*N* = 32)**.

**Figure 7:**
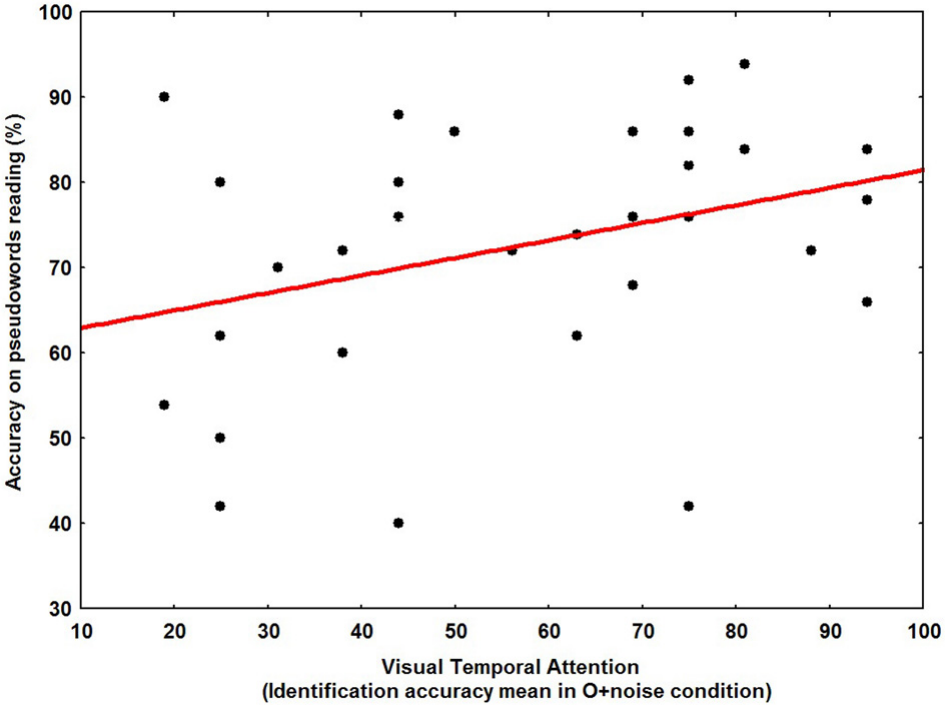
**Scatter plot of the relationship between visual temporal attention (identification accuracy mean in O+noise condition) and pseudoword reading accuracy across our entire sample of dyslexic children (*N*= 32)**.

The main results showed that phonological decoding was significantly correlated with both spatial and temporal attention as well as phoneme blending (see Figures 6, 7). In addition, irregular word reading was correlated with both phonological tasks and VSA. Finally, temporal and spatial attention were highly correlated to each other (see Table 5A). The same bivariate correlations were computed in our entire sample (*N* = 75), including the NR children, confirming the relationship between visual attention and phonological decoding (see Table 5B).

**Table 5:** **Bivariate correlations between abilities were measured on Regular and Irregular words and Pseudowords, time-course of visual spatial attention (VSA; RT difference between cuing effect at 350 and 100 ms SOA), time-course of visual temporal attention (VTA; identification accuracy mean in O+noise condition), pseudowords phoneme blending (number of correct pseudowords), and a pseudoword short term memory (number of correct phonemes)**.

	**Regular words reading (%)**	**Irregular words reading (%)**	**Pseudowords reading (%)**	**Number of correct pseudowords**	**Number of correct phonemes**	**VTA**
**(A)**						
Irregular words reading (%)	0.937^**^	–				
Pseudowords reading (%)	0.691^**^	0.739^**^	–			
Number of correct pseudowords	0.336	0.436^*^	0.463^**^	–		
Number of correct phonemes	0.172	0.350^*^	0.159	0.574^**^	–	
VTA	0.056	0.120	0.333^*^	−0.063	0.012	–
VSA	−0.322	−0.374^*^	−0.476^**^	−0.239	−0.325	−0.517^**^
**(B)**						
Irregular words reading (%)	0.932^**^	–				
Pseudowords reading (%)	0.717^**^	0.743^**^	–			
Number of correct pseudowords	0.430^**^	0.502^**^	0.543^**^	–		
Number of correct phonemes	0.301^**^	0.439^**^	0.372^**^	0.613^**^	–	
VTA	0.117	0.206	0.310^**^	0.168	0.181	–
VSA	−0.267^**^	−0.347^**^	−0.267^*^	−0.203	−0.255^*^	−0.295^*^

*Bivariate correlations computed in dyslexic children (N = 32) are reported in the **A**. Bivariate correlations computed in the entire sample (N = 75) are reported in the **B***.

* *Correlation is significant at the 0.05 level*.

** *Correlation is significant at the 0.01 level*.

To determine predictive relationships between visual attention and reading (pseudoword and irregular) accuracy, we computed two four-step fixed-entry multiple regression analyses on the individual data of the dyslexic children to control for the effects of age, Performance IQ, attentional mechanisms, and phonological processing.

Descriptive statistics of variables included in the multiple regression analysis are reported in Table 6. Because attentional graphemic parsing precedes phonemic blending during the reading process and VSA precedes the perceptual noise exclusion mechanism, we selected the following order of steps. In the first multiple regression analysis the dependent variable was pseudoword reading accuracy and the predictors entered at the four steps were: (i) age and Performance IQ, (ii) VSA, (iii) VTA, and (iv) PPB. Results showed that the time-course of spatial attention accounted for 17% (*p* < 0.02) of unique variance in sub-lexical reading accuracy. Only the PPB entered at the last step accounted for 14% (*p* < 0.05) of unique variance in lexical reading accuracy (see Table 7).

**Table 6:** **Descriptive Statistics of variables included in the multiple regression analysis: age (month), Performance IQ (Figure Completion, Wechsler, 1993), pseudoword reading accuracy, time-course of visual spatial attention (VSA; RT difference between cuing effect at 350 and 100 ms SOA) and time-course of visual temporal attention (VTA; identification accuracy mean in O+noise condition), and pseudowords phoneme blending (PPB; number of correct pseudowords) in dyslexic children (*N* = 32)**.

	**DD (*N* = 32)**	
	***M***	***SD***
Age (months)	122.53	22.16
Performance IQ (ss)	12.53	3.02
Pseudowords reading (%)	72.5	14.70
VSA	13.81	79.50
VTA	56.59	23.84
Number of correct phonemes	38.53	14

**Table 7:** **Multiple regression analysis with pseudoword reading accuracy as dependent variable and the following predictors entered at the four steps: (i) age and Performance IQ, (ii) time-course of visual spatial attention (VSA; RT difference between cuing effect at 350 and 100 ms SOA), (iii) time-course of visual temporal attention (VTA; difference between signal accuracy and signal + noise accuracy), and (iv) pseudowords phoneme blending (PPB; number of correct pseudowords) in dyslexic children (*N* = 32)**.

**Predictors**	***R***	***R*^2^**	**Change Statistics**				
			***R*^2^ Change**	***F* Change**	***df1***	***df2***	**Sig. *F* Change**
Age and performance IQ	0.283	0.080	0.080	1.265	2	29	>0.05
VSA	0.501	0.251	0.171	6.380	1	28	**<0.02**
VTA	0.506	0.256	0.005	0.187	1	27	>0.05
PPB	0.628	0.394	0.138	5.908	1	26	**<0.05**

In the second multiple regression analysis the dependent variable was irregular word reading accuracy and the predictors entered at the four steps were the same as in the first analysis. Only the PPB entered at the last step accounted for 12% (*p* < 0.05) of unique variance in lexical reading accuracy (see Table 8).

**Table 8:** **Multiple regression analysis with irregular word reading accuracy as the dependent variable and the following predictors entered at the four steps: (i) age and Performance IQ, (ii) time-course of visual spatial attention (VSA; RT difference between cuing effect at 350 and 100 ms SOA), (iii) time-course of visual temporal attention (VTA; difference between signal accuracy and signal + noise accuracy), and (iv) pseudowords phoneme blending (PPB; number of correct pseudowords) in dyslexic children (*N* = 32)**.

**Predictors**	***R***	***R*^2^**	**Change Statistics**				
			***R*^2^ Change**	***F* Change**	***df1***	***df2***	**Sig. *F* Change**
Age and performance IQ	0.266	0.071	0.071	1.102	2	29	>0.05
VSA	0.402	0.162	0.072	0.091	1	28	>0.05
VTA	0.438	0.192	0.030	1.006	1	27	>0.05
PPB	0.556	0.309	0.117	4.381	1	26	**<0.05**

## Discussion

Our results demonstrate that both spatial and temporal attention were impaired only in dyslexics with a poor phonological decoding (DDP−), confirming the relationship between visual attentional mechanisms and graphemic parsing processes. It is important to note that attentional graphemic parsing precedes the letter-to-speech sound integration.

The attentional cuing effect was present at the shortest cue-target delay (100 ms) in both NR and DDP+, as predicted by automatic capture theories (for a review, see Klein, 2000). In contrast, DDP− children showed a slower visual-spatial attentional orienting, because the cuing effect was not present at the short cue-target delay (distributed attention) whereas it appears at the long cue-target delay, as predicted by the “SAS” theory (Hari and Renvall, 2001; Facoetti et al., 2010a; Lallier et al., 2010; see Facoetti, 2012, for a recent review). We note that in DDP− the cuing effect was stronger than in DDP+ and NR grouped together at the long cue-target delay, suggesting that poor phonological decoders present a more concentrated focus of attention on the cue. These findings could reconcile apparent contradictory results in the literature regarding the different size of the attentional focus in dyslexics. For example, according to some evidence the attentional focus appears more distributed in dyslexics in comparison to normally reading children (e.g., Geiger et al., 1994, 2008; Facoetti et al., 2000). In contrast, other studies have shown more focused attention in dyslexics in comparison to normally reading children (e.g., Bosse et al., 2007). In general, before the cue onset, the attentional focus—controlled by the right frontal eye fields (Ronconi et al., 2014)—is probably distributed across the possible target locations indicated by the two circles. The attentional focus of the poor phonological decoders will be on the cue location for long cue-target delay (cuing effect), whereas their attentional focus will not be there for short cue-target delay (absence of cuing effect), suggesting a sluggish attentional orienting (Facoetti et al., 2010a; see Hari and Renvall, 2001; Facoetti, 2012, for reviews). Once on the cue, the attentional focus appears more focused in comparison to the other two groups. This combination of spatial attention disorders probably impairs the serial letter processing during graphemic parsing.

Moreover, object identification in the object task without noise was not impaired in poor phonological decoders, excluding a possible general visual perception deficit. In contrast, DDP− showed a specific identification deficit in comparison to NR and DDP+ when the object was displayed with noise (i.e., masks and a second object), demonstrating an inefficient perceptual-noise exclusion mechanism. Although the DDP− group showed a larger second object substitution, it would be important for future research to incorporate a baseline measure including O1+mask, but excluding O2, which would better isolate the role of specific O2 interference on O1. This condition could be relevant to even better isolate the pure role of the O2 interference, otherwise it would be difficult to exclude that unknown processing speed differences between the groups may play a role in the results.

According to the phonological hypothesis, it is important to note that poor pseudoword reading accuracy is strongly related to impaired phonological awareness (Frith, 1997; Snowling, 2000; Goswami, 2003, 2011; Vellutino et al., 2004). However, the two samples of dyslexics were not different in general phonological processing (i.e., phoneme blending and short-term memory of pseudowords). Only in DDP− both spatial and temporal attentional tasks were specifically disturbed, consistent with the hypothesis that SAS (Hari and Renvall, 2001) contributes to difficulties in phonological decoding, and it is at least partially independent from phonological skills (Bosse et al., 2007; Facoetti et al., 2010a,b; Franceschini et al., 2012, 2013; Zorzi et al., 2012; see Vidyasagar and Pammer, 2010, for a review).

Our attention indices allowed us to discriminate between dyslexics with poor phonological decoding and dyslexics with unimpaired phonological decoding or normal readers. Our results suggest that visual attention impairments are the core deficit in dyslexics characterized by poor (i.e., inaccurate) phonological decoding. This finding was supported by the predictive relationship of reading performance and visual attentional tasks, even after controlling for age and Performance IQ. Attentional graphemic parsing was significantly related to phonological decoding because it represents the first step that precedes not only letter-to-speech sound integration but also phonemic blending (significantly related to pseudoword reading) during the reading process.

It is important to stress that the predictive relationship between attention and reading skills held across the entire sample of dyslexics, independently of any a priori classification or subtyping of the dyslexic children. Thus, regardless of whether children in the DDP+ group constitute a specific subtype in shallow orthographies (Wimmer, 1993) or have partly compensated their reading deficit, rapid and efficient orienting of spatial attention seems to be related to phonological decoding. We suggest that this relationship might be causal because: (i) VSA is impaired in preschoolers at risk of DD (Facoetti et al., 2010b); (ii) it represents a significant predictor of future reading abilities (Franceschini et al., 2012); and (iii) attentional video games training has been proven to increase reading skills (Franceschini et al., 2013).

Our findings are consistent with previous results (e.g., Roach and Hogben, 2007; Facoetti et al., 2010a), and with the predictions of the CDP+ computational model of reading aloud (Perry et al., 2007). Efficient focused attention—indicated by a cuing effect at the short cue-target delay—is necessary for serial letter processing during phonological decoding, limiting the perceptual noise. In the DDP− group the cuing effect was absent at short cue-target delay, increasing the interference produced by perceptual noise during letter processing. Accordingly, several studies suggest that a general disorder in ignoring task-irrelevant information characterizes dyslexia perceptual processing (e.g., Badcock et al., 2008, 2011; Roach and Hogben, 2008). The sluggish attentional orienting index (spatial attention, task 1) is linked to a perceptual noise exclusion mechanism (temporal attention, task 2). The CDP+ assumes that focused attention is specifically involved in the sub-lexical spelling-to-sound mapping process (i.e., the sub-lexical route). Visual attentional tasks accounted almost for 20% of unique variance in phonological decoding, representing an excellent predictor of pseudoword reading. Moreover, irregular word reading accuracy was not significantly predicted by the visual attentional variables, but only by the phonological ones (which accounted for 12%).

Clearly, these results are inconsistent with the hypothesis that DD is an exclusively phonological deficit. The present link between deficits in spatial and temporal attention and impaired phonological decoding is consistent with the hypothesis that visual selection (i.e., the perceptual-noise exclusion mechanism) operates on graphemes as the basic component of the phonological assembly process (Cestnick and Coltheart, 1999; Perry et al., 2007, Gori et al., under revision in the same issue). Both spatial (Geiger and Lettvin, 1987; Sperling et al., 2005; Geiger et al., 2008) and temporal (Di Lollo et al., 1983; Visser et al., 2004; Montgomery et al., 2005; Facoetti et al., 2008) processing windows in which noise interferes with the signal appear to be broader in dyslexics than normally reading children. In this study, we demonstrated that these deficits are specific in poor phonological decoders, and this can be attributed to the perceptual-noise exclusion deficit (Sperling et al., 2005). The link between deficits in VSA and impaired phonological decoding is also consistent with the results of recent studies in dyslexics that used visual search paradigms (e.g., Buchholz and McKone, 2004; Roach and Hogben, 2007; Jones et al., 2008; Facoetti et al., 2010a; Vidyasagar and Pammer, 2010, for a review). Furthermore, our results demonstrate, for the first time, that the relationship between visual attention and phonological decoding skills in dyslexia is explained by a sluggish shifting of spatial attention rather than a general perceptual noise exclusion mechanism. We suggest that inefficient spatial attention could specifically impair the graphemic parsing mechanism in dyslexic children. Although our spatial attention task involves also a temporal component, several studies have shown that the rapid shift of spatial attention modulates the speed of processing and consequentially the temporal aspects of attention (e.g., Carrasco and McElree, 2001; Carrasco et al., 2002, 2004; see Enns and Di Lollo, 2000, for a review). However, further studies are necessary to investigate the specific relationship between spatial and temporal attention. The results of the present study do not speak to the issue of visual vs. auditory and phonological processing deficits in DD. Several authors have argued that the core problem in DD is a deficit in phonological representation (Snowling, 2000; Ramus, 2003). It is important to note that efficient learning of sub-lexical spelling-sound mappings requires not only graphemic parsing but also accurate auditory and speech-sound segmentation mechanisms (see Goswami, 2003, 2011, for reviews). In particular, rise times are crucial events in the speech signal, as they reflect the patterns of amplitude modulation that facilitates the temporal segmentation of the acoustic signal into syllables. Rise time discrimination is impaired in dyslexia in English, French, Hungarian, Spanish, Chinese, and Finnish (Goswami, 2011). Rise time is a significant predictor of phonological awareness. However, efficient acoustic processing and segmentation of the speech signal are likely to require the rapid engagement of auditory attention (Renvall and Hari, 2002; Facoetti et al., 2003, 2005, 2010a). Auditory attention is, indeed, necessary for speech segmentation based on statistical learning (Toro et al., 2005) and for learning phonetic discriminations based on acoustic cues (Gordon et al., 1993; Francis et al., 2008; but see also Seitz et al., 2010). Moreover, auditory spatial attention has been shown to be defective in children with specific language impairment (SLI; Stevens et al., 2006) and reading DD (Asbjørnsen and Bryden, 1998; Renvall and Hari, 2002; Facoetti et al., 2003, 2005, 2010a,b).

Neural coding by brain oscillations is a major focus in neuroscience (e.g., Buzsaki and Draghun, 2004; Schroeder et al., 2008), with important implications for DD research (see Goswami, 2011, for a recent review). The results could be interpreted inside the oscillatory “temporal sampling” framework which is a compelling and robust theoretical framework (Goswami, 2011). Temporal sampling of speech-sound by neuroelectric oscillations that encode incoming information at different frequencies could explain the perceptual and phonological difficulties with syllables, rhymes and phonemes found in individuals with DD. A temporal sampling framework based on oscillations that entrain to sensory input could also have implications for other sensory theories of DD such as the magnocellular-dorsal (M-D) deficit theory (see Stein and Walsh, 1997; Gori and Facoetti, 2013, for a recent review). Thus, we conclude that a temporal sampling disorder of neural oscillations could characterize DD, suggesting innovative training programs not only for treatment but also for the possible prevention of DD at the pre-reading stage.

### Conflict of interest statement

The authors declare that the research was conducted in the absence of any commercial or financial relationships that could be construed as a potential conflict of interest.

## Footnotes

^1^Italian irregular words are defined as words stressed at third or fourth syllable from last (e.g., rùvido, dòllaro, àbitano, dèlegano).

^2^The main effect of the O1 pre-mask variable time exposure (175, 225, 275 or 325 msec) and the interaction effect with the group were not significant (*p*s > 0.05).
