# Supplementary material for: Spatial and temporal attention in developmental dyslexia
Source: Front Hum Neurosci. 2014 May 22;8:331. doi: 10.3389/fnhum.2014.00331 (PMC4033052; doi:10.3389/fnhum.2014.00331)
Supplement: Supplementary file 1 [file DataSheet1.DOCX]

**SUPPLEMENTARY MATERIAL**

Pseudowords Phoneme Blending (PPB) task

gastibo

nositra

lonteski

grilandi

nastabi

spodarti

lemorghi

komudri

tedonka

kinupentro

Pseudoword Short Term Memory (PSTM) task

| 2 | sed-gam  tul-sid |
| --- | --- |
| 3 | fib-nup-gan  rag-bil-sut |
| 4 | tol-vus-rab-dig  cal-ner-dig-bov |
| 5 | tuf-sev-gal-cid-fom  bes-rad-niv-cot-puc |
| 6 | saz-vum-tob-nic-rel-fup  lem-gor-vus-rab-tin-fad |
| 7 | sat-mid-bog-tur-dab-fip-zal  nir-cov-pef-ghip-das-lon-tif |
| 8 | bor-cit-vem-fal-saf-cub-nid-pog  teb-pid-mac-nor-ked-faz-sel-vup |
